# CX3CL1/CX3CR1 in Alzheimer's Disease: A Target for Neuroprotection

**DOI:** 10.1155/2016/8090918

**Published:** 2016-06-27

**Authors:** Peiqing Chen, Wenjuan Zhao, Yanjie Guo, Juan Xu, Ming Yin

**Affiliations:** ^1^School of Pharmacy, Shanghai Jiao Tong University, 800 Dongchuan Road, Shanghai 200240, China; ^2^Department of Neurology, Shanghai General Hospital, Shanghai Jiao Tong University School of Medicine, No. 100 Haining Road, Shanghai 200080, China

## Abstract

CX3C chemokine ligand 1 (CX3CL1) is an intriguing chemokine belonging to the CX3C family. CX3CL1 is secreted by neurons and plays an important role in modulating glial activation in the central nervous system after binding to its sole receptor CX3CR1 which mainly is expressed on microglia. Emerging data highlights the beneficial potential of CX3CL1-CX3CR1 in the pathogenesis of Alzheimer's disease (AD), a common progressive neurodegenerative disease, and in the progression of which neuroinflammation plays a vital role. Even so, the importance of CX3CL1/CX3CR1 in AD is still controversial and needs further clarification. In this review, we make an attempt to present a concise map of CX3CL1-CX3CR1 associated with AD to find biomarkers for early diagnosis or therapeutic interventions.

## 1. Introduction

Alzheimer's disease (AD), a common progressive neurodegenerative disease, is the most frequent cause of cognitive decline and dementia, which affects more than 46 million people worldwide. The etiology of AD is still unclear now. One of the main pathological characteristics is extracellular deposits of *β*-amyloid (A*β*) peptides in senile plaques. A*β* cascade-inflammatory hypothesis has been elucidated to look forward to seeking treatment for AD [1]. Some scholars believe that A*β*-burdened neurons may play a crucial role in initiating microglial activation and eliciting chronic inflammation which lead to synaptic dysfunction, neurotoxicity, and behavioral deficits in the progression of AD [2–6]. Reactive microglia is also related to driving tau pathology and correlating with the spread of tau pathology [7], which induces neurofibrillary tangles (NFT), another major pathological characteristic of AD. Consistently, depleting microglia dramatically suppressed the propagation of tau in the brain [8].

CX3C chemokine ligand 1 (CX3CL1, also named fractalkine) plays an important role in reducing neuroinflammation and is highly expressed in the main area of pathological changes in AD, such as the hippocampus and cerebral cortex, and the expression level of CX3CL1 reflects the progression of the disease [9]. CX3CL1 has been demonstrated to play a neuroprotective role in CNS by reducing neurotoxicity and microglial activation [10–12]. Consistent with this is the fact that treatment of aged rats with CX3CL1 attenuates the age-related increase in microglial activation [13]. Moreover, CX3CL1 also has an effect on A*β* clearance and p-tau accumulation in AD [14]. All the above show that CX3CL1 has a major role in the progression of AD. In this review, we summarize the multiple roles of CX3CL1 in neuroinflammation, neurotoxicity, and synaptic plasticity in AD pathogenesis.

## 2. CX3CL1/CX3CR1 and Microglia

CX3CL1 is a large cytokine protein of 373 amino acids with an extended mucin-like stalk and a chemokine domain on top. It is the only member of CX3C family which belongs to the large family of small secreted chemotactic cytokines. CX3CL1 is expressed with particularly high levels in hippocampal and cortical neurons constitutively but none on microglia [15]. It exists in both secreted and membrane-bound form and its membrane-tethered mucin stalk acts as a cell adhesion molecule adhering to microglia during an inflammatory reaction [16]. The membrane-bound form can be cleaved in the condition of cathepsin S, ADAM-10, and ADAM-17; then the soluble one can serve as a signaling molecule mediating neural/microglial interactions via its sole receptor CX3CR1 that is mainly expressed on microglia and partly on astrocyte as well as on neurons in the CNS [17–19]. These suggest that CX3CL1/CX3CR1 is an important bridge to connect neuron and microglia.

Microglia, resident mononuclear phagocytes in the CNS, intimately involved in the development of the nervous system, are highly active in their presumed resting state, continually surveying their microenvironment with extremely motile processes and protrusions [20, 21]. It has been demonstrated that A*β* burdened neurons inducing microglial activation may be an early phenomenon in the procession of AD [22]. However, microglia activation in AD is suggested to be heterogeneous: beneficial or harmful [23]. This may be associated with microglia activation phenotype which includes M1 (iNOS^+^ microglia) and M2 (Arg^+^ microglia); iNOS^+^ microglia induce production of neuroinflammation factors while Arg^+^ microglia have enhanced phagocytic activity. In accordance with this, greater numbers of Arg^+^ microglia containing A*β* were found when compared to iNOS^+^ microglia in the inflamed hemisphere [24]. Moreover, amounts of evidence indicate that microglia phenotype changes from M2 to M1 in the progression of AD [25].

Neuronal soluble CX3CL1 is likely to alter the microglial state to a more neuroprotective one by acting on CX3CR1 in microglia [26]. This also has been confirmed that disruption of CX3CL1-CX3CR1 leads to dysregulate microglial responses and neuronal damage [12, 18]. Besides, hAPP-CX3CR1^−/−^ mice as well as hTau-CX3CR1^−/−^ mice showed increased expression of inflammatory factors, enhanced tau phosphorylation, and exacerbated plaque-independent neuronal dysfunction and cognitive deficits [27, 28], while researches also demonstrated that both APP-PS1/CX3CR1^−/−^ and CRND8/CX3CR1^−/−^ mice showed reduction in A*β* deposition with increased number of microglia [29, 30]. Moreover, the suppression of CX3CL1-CX3CR1 alleviated A*β*-induced neurotoxicity and memory deficiency [31, 32]. Well, CX3CL1/CX3CR1 may play a beneficial role in controlling the progression of AD by inhibiting the inflammation and tau phosphorylation but at a cost of the increased A*β* deposition. Overexpression of soluble CX3CL1 by adeno-associated viral (AAV) vectors plays an active role in reducing tau pathology and neuron loss, while it has no effect on A*β* deposition indicating that additional CX3CL1 signaling has no additive effect on A*β* deposition [26, 33]. Surprisingly, neither enhanced tau phosphorylation nor reduced A*β* deposition in CX3CL1-deficient APP-PS1 animals was altered by soluble CX3CL1 isoform, which was introduced by bacterial artificial chromosome (BAC) transgene encoding truncated CX3CL1 [34]. Thus making the function of soluble CX3CL1 is full of doubt. A possible explanation is that AAV vectors might make soluble CX3CL1 build the required local gradient and it should suffice, while the only soluble CX3CL1 can be diluted rapidly [35]. This needs to be further clarified.

The expression of CX3CL1 is decreased in cerebral cortex and hippocampus of APP transgenic mice while it is increased in tau-injured neurons [36, 37]. Moreover, the level of plasma soluble CX3CL1 is significantly greater in the patients with mild to moderate AD than in the patients with severe AD, and the level of CX3CL1 is inversely correlated to AD severity [38]. Together, these studies suggest that CX3CL1/CX3CR1 associated with neuroinflammation, neurotoxicity, and synaptic plasticity plays variable roles in different stages of AD pathogenesis. Considering this, we conjecture that mild decreased CX3CL1-CX3CR1 due to intraneuronal A*β* accumulation in the early stage of AD increases clearance of A*β* deposition by enhancing the phagocytosis of microglia while resulting in tau hyperphosphorylation and severe downgraded CX3CL1-CX3CR1 signal gives rise to deregulated microglia and abnormally excited neuron which lead to neuron damage and loss in the progression of AD.

## 3. CX3CL1/CX3CR1 and Neuroinflammation

Neuroinflammation is classically attributed to A*β* deposition and plays a vital role in the pathological progress of AD [5, 39]. It is always correlated with increased levels of proinflammatory cytokines including tumor necrosis factor-alpha (TNF-*α*), interleukin-6 (IL-6), IL-1*β*, interferon gamma (IFN-*γ*), and chemokine (C–C motif) ligand 2 (CCL2) and C–X–C motif chemokine 10 (CXCL10/IP-10) [40]. CX3CL1, which is identified inhibiting the production of TNF-*α*, nitric oxide (NO), and superoxide in neuron-glial cell cultures [41], has been implicated as an endogenous neuronal modulator and may limit microgliosis in AD by reducing the inflammatory reaction [37, 42, 43].

TNF-*α*, a prototypic proinflammatory cytokine, is mainly released by activated microglia, colocalized with A*β* deposition, and is elevated in the cortex of animal models and human with AD [44–46]. It has been shown that glial TNF-*α* enhances A*β* deposition through inhibiting BACE1 expression and A*β* clearance and promotes neuronal cell cycle events which are toxic for terminally differentiated neurons in the pathogenesis of AD [47, 48]. Besides, Lourenco et al. have proved that A*β* oligomers lead to synapse loss and memory impairment in a TNFR1 dependent manner [49]. TNF-*α* actives TNFR1 leading to neuron death while TNFR2 which is expressed primarily by microglia [50] is beneficial to control microglia activity in the progression of AD [51].

Fewer A*β* plaques and A*β*-related lesions developed in APP23/TNFR1^−/−^ mice when compared with APP23/TNFR1^+/+^ littermates [52]. However, Barger et al. suggested that TNF-*α* protects hippocampal neurons against A*β* toxicity [53]. Both 3xTg-AD lacking TNF-R1+R2 and 3xTg-ADxTNF-R1/R2 knock-out exhibit enhanced A*β* and tau-related pathological features by the age of 15 months, in stark contrast to age-matched 3xTg-AD counterparts [54]. Loss of opposing TNFR2 leads to a stage-independent increase in Iba-1 positive microglia, and TNFR1 mediated exacerbation of A*β* and tau pathology in aged 3xTg-AD mice [55]. Thus suggesting the role of CX3CL1/CX3CR1 which inhibits TNF-*α* secretion [56] may be divaricated dependent on TNFR. But in view of the fact that TNFR1 is increased by 17–28% and TNFR2 is significantly decreased by 35–43% in AD brains [57], CX3CL1/CX3CR1 inclines to play a beneficial role in the pathogenesis of AD.

The expression of another inflammatory cytokine IL-1*β* is also increased in the CX3CR1-deficient APP/PS1 animals [29]. The major role of increased IL-1*β* in neuroinflammation and subsequent induction of the microglial autophagy potentially are contributed to AD [58, 59]. CX3CR1 deficiency promotes impairment of cognitive function, synaptic plasticity, and tau hyperphosphorylation via increasing action of IL-1*β* and the impairment could be reversed by infusion with IL-1*β* receptor antagonist significantly [28, 42]. On the other hand, the upregulated expression of chronic IL-1*β* increases plaque-associated microglia and ameliorates amyloid pathology in the APP/PS1 mouse model of AD [60, 61]. The generation of this contradiction is likely to depend on the stage of AD, which may be coordinated with CX3CL1 functions in different period.

In addition, CX3CL1 dose-dependently suppressed the production of nitric oxide (NO) [10]. NO, related to the increased levels of IFN-*γ* and TNF-*α* [62], has been involved in neuroinflammation with increased expression of inducible NO synthase (iNOS) at mild and severe stages of AD [63]. Inhibition of iNOS which mediates CNS inflammatory processes reduces the risk of AD [64]. In all, CX3CL1-CX3CR1 inhibits microglia activity via controlling the overproduction of inflammatory mediators. The distinctly decreased expression of CX3CL1 gives rise to dysregulated microglia, leading to neuroinflammation. Drugs that attenuate neuronal degeneration and improve learning and memory ability are accompanied by reduced TNF-*α*, IL-1*β*, TGF-*β*, and NO levels induced by A*β* in CSF in mouse models and patients with AD [65–70]. Apart from AD, CX3CL1/CX3CR1 is also involved in other neuroinflammation disorders, including Parkinson's Disease (PD) [71, 72], multiple sclerosis (MS) [73], tauopathies [33], and age-related macular degeneration (ARMD) [74]. These neurodegenerative disorders are all associated with chronic neuroinflammation caused by activated microglia [75], indicating that CX3CL1/CX3CR1 may have the similar mechanisms between AD and other neurodegenerative disorders in regulating neuroinflammation. The complex roles of CX3CL1/CX3CR1 are still being studied.

## 4. CX3CL1/CX3CR1 Regulates Synaptic Plasticity

Synaptic plasticity plays an important role in learning and memory, and A*β*-induced synaptic dysfunction is strongly associated with AD [76]. CX3CL1 is upregulated in the rat hippocampus during memory-associated synaptic plasticity [77]. It is considered as a potent neuromodulator of the evoked excitatory synaptic transmission and plays a major role in synaptic plasticity and neuroprotection [78]. Furthermore, the functions of CX3CL1 rely on CX3CR1, as long-lasting-enriched environment failed to affect hippocampal-dependent plasticity in the absence of CX3CR1 [79]. Although the underlying mechanisms have been underexplored, CX3CL1/CX3CR1 may mediate synaptic plasticity and cognitive function mainly by regulating long-term potentiation (LTP) [80], NO signaling, and production of brain-derived neurotrophic factor (BDNF) [81].

LTP is thought to be related to the storage of declarative memory in the mammalian brain [82]. CX3CL1 clearly interferes with LTP mechanisms and its modulation of neuronal plasticity appears to be mediated through activation of adenosine [80]. Adenosine acts as a neuromodulator with four types of G protein-coupled receptors, termed A1, A2A, A2B, and A3, and exerts important functions in the synaptic plasticity [83]. The downstream pathways branch because of the different types of adenosine receptor. Intracerebroventricular injection of A*β*_1–42_ inhibited not only NMDA receptor-dependent LTP but also voltage-activated Ca^2+^-dependent LTP induced by strong conditioning stimulation during NMDAR blockade [84], indicating that there is a non-NMDAR-dependent but Ca^2+^-dependent pathway involved in synaptic dysfunction in AD. CX3CL1 increases NMDA-fast excitatory postsynaptic potentials by a mechanism involving the activity of the adenosine receptor type A2 (A2AR) and the release of the NMDAR coagonist D-serine [85]. NMDAR activation affects the threshold for LTP induction which is strongly influenced by the recent history of synaptic activity [86]. An increased density of A2AR on microglia has been detected in human cortex from AD patients [87]. Thus indicating CX3CL1/CX3CR1 may activate A2AR by increasing adenosine and promote the release of D-serine; then D-serine enhances the function of NMDAR and facilitates LTP. Moreover, CX3CL1 causes a reversible depression of excitatory postsynaptic current (EPSC), which is abolished by the A3R antagonist [88], and the inhibition failed to occur in CX3CR1 null mice [80]. Stimulation of A3R induces an intracellular signaling that increases calcium concentrations [89]. The phosphorylation of CAMKII and cyclic adenosine monophosphate response element-binding protein (CREB) is important to hippocampal long-term synaptic plasticity [90]. *α*CaMKII autophosphorylation is also required for synaptic plasticity induced by a short and precise stimulus, but maybe not for a longer and stronger stimulation [91]. Besides, the reduction of CREB activation also leads to memory impairment [92]. Based on the information given above, we can hypothesize the way CX3CL1 affects LTP; that is, CX3CL1 acts with CX3CR1 on the surface of the microglia and stimulates the release of adenosine; adenosine then activates A2AR and promotes synaptic facilitation by NMDAR-dependent pathway, activates A3R simultaneity, and induces synaptic inhibition by a Ca^2+^-dependent pathway.

Brain-derived neurotrophic factor (BDNF), an important growth factor in the CNS, is of great significance for neurons to maintain the survival, growth, differentiation, repair, and regeneration after nerve injury as well as increasing synaptic plasticity. A clinical study involving 535 old participants who underwent annual cognitive assessments and brain autopsy at death showed that higher brain BDNF expression is associated with slower cognitive decline and BDNF may also reduce the deleterious effects of AD pathology on cognitive decline [93]. Studies have shown that A*β* induces decreased anterograde as well as retrograde transport of BDNF vesicles in hippocampal neurons of various AD models [94]. Upregulation of BDNF by activating of ERK/CREB pathway can ameliorate A*β*-induced neurons loss and dendritic atrophy [95]. Restoration of normal neuronal BDNF expression levels in the cerebral hippocampi and cortices ameliorates the impairment in recognition memory and associative learning in mice of AD [96]. Importantly, BDNF concentrations are associated with CX3CL1 [97]. Chronic injection of CX3CL1 rescues the hippocampal-dependent memory deficits and reverses the decreased hippocampal neurogenesis in genetic BDNF variation mice [98].

In addition, NO is also consistently involved in recognition memory [99]. As mentioned before, CX3CL1/CX3CR1 inhibits the expression of NO in activated microglia cells [10, 100] and may induce synaptic inhibition. NO signaling through neuronal NO synthase (nNOS) prior to the appearance of cognitive symptoms focuses on early developments of AD [63]. NO/soluble GC (sGC)/cGMP-PKG and ERK signaling is important for modulating synaptic transmission and plasticity in the hippocampus and cerebral cortex, which are critical for learning and memory [101]. Recruitment of NO is serving a compensatory role to boost synaptic transmission and plasticity during early AD stage [102]. NO inhibitors ameliorate overexpressed NMDA receptor subunit NR2B which plays a role in memory formation in an inflammatory model of AD [103]. Besides, endothelial NO deficiency also promotes AD pathology [104].

## 5. CX3CL1/CX3CR1 Reduces Excitotoxicity

It has been verified that CX3CL1 released from hippocampal cells after excitotoxic insult has an essential role in brain protection by reducing against glutamate mediated excitotoxicity [105]. As mentioned before, microglia shape their neuronal environment actively thanks to their ability to trigger neuronal death [106–108]. Apart from regulating neuroinflammation, CX3CL1/CX3CR1 negatively modulates the function of AMPA receptor at active glutamatergic synapses [109]. CX3CL1 reduces the glutamate mediated excitotoxicity by reducing the influx of Ca^2+^ [105]. Calcium channel blockers also exhibit cognitive enhancing abilities and reduce the risk of dementia genuinely [110]. Moreover, the application of ion channel blockers with specific antagonists of the NR2B subunit could reduce neurotoxicity significantly [111]. Besides, CX3CL1 mediated neuroprotection by increasing glutamate transporter-1 (GLT-1) activity on astrocytes is dependent on the presence and the activity of A1 adenosine receptor (A1R), which can be blocked by the specific antagonist DPCPX and absent in A1R^−/−^ astrocytes [112, 113]. Consistently, hippocampal neurons obtained from A1R^−/−^ mice are not protected by CX3CL1 against Glu excitotoxicity [114]. Collectively, these data indicate that CX3CL1/CX3CR1 reduces excitotoxicity by modulating glutamatergic transmission and may play an important role in cognitive functions in AD.

## 6. Conclusions and Perspectives

**Figure 1:**
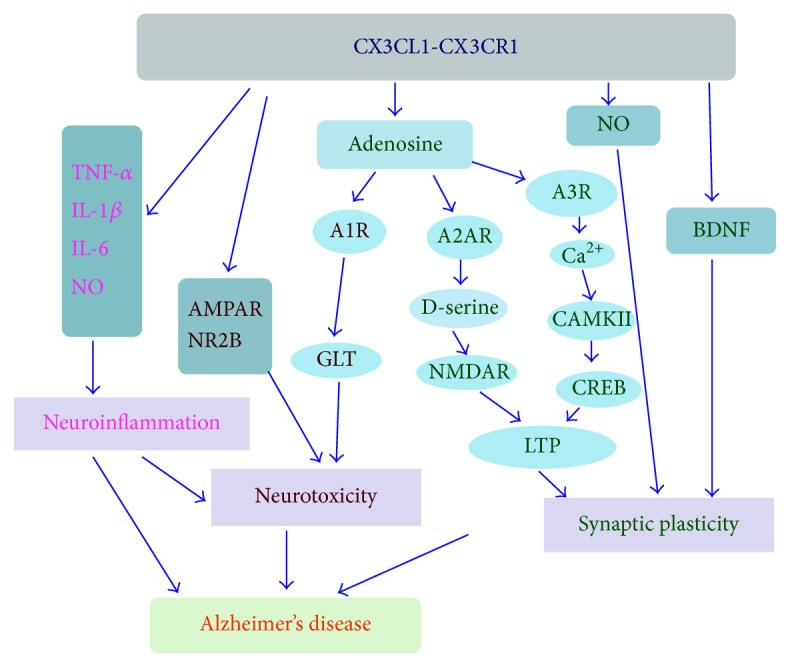
The effect of CX3CL1/CX3CR1 in Alzheimer's disease (AD). CX3CL1 binds to CX3CR1 which is its sole receptor and mainly expressed by microglia: (1) regulating introduction of inflammation cytokine (TNF-*α*, IL-1*β*, IL-6, NO, etc.) and reducing neuroinflammation in AD; (2) negatively modulating the function of AMPAR and NR2B, increasing GLT activity through the mechanism dependent on A1R, and then decreasing the neurotoxicity induced by Glu; (3) stimulating the release of adenosine; adenosine then activates A2AR and promotes synaptic facilitation by NMDAR-dependent pathway and simultaneity activates A3R and induces synaptic inhibition by Ca^2+^-dependent pathway. TNF-*α*: tumor necrosis factor-alpha; IL-1*β*: interleukin-1*β*; IL-6: interleukin-6; NO: nitric oxide; A1R: adenosine 1 receptor; A2AR: adenosine A2a receptor; A3R: adenosine 3 receptor; GLT: glutamate transporter; LTP: long-term potentiation; CREB: cyclic adenosine monophosphate response element-binding protein; BNDF: brain-derived neurotrophic factor.

There is persistent neuroinflammation throughout the progression of AD associated with neurotoxicity and synaptic dysfunction [115, 116]. The expression of CX3CL1 is significantly decreased in AD and inversely correlated to AD severity. As shown in Figure 1, CX3CL1/CX3CR1 may regulate the activation of microglia by controlling the release of inflammatory cytokines and synaptic plasticity and cognitive functions by modulating receptors in neurons directly or indirectly. The involvement of CX3CL1/CX3CR1 in AD suggests that CX3CL1/CX3CR1 contributes positively to neuron protective as well as detrimental role in the course of the disease. Therefore, targeting CX3CL1 and/or CX3CR1 may provide novel opportunities for treatment of AD. In particular, the development stage of the disease should be considered to better analyze the functions of CX3CL1/CX3CR1 in the progression of AD.

In addition, experiment evidences have described the active involvement of CX3CL1/CX3CR1 in many other diseases, such as atherosclerotic, allergic asthma and rhinitis, renal diseases, rheumatoid arthritis (RA), Sjögren's syndrome (SS), systemic lupus erythematosus (SLE), scleroderma, colorectal cancer, and breast cancer [117–123]. For example, a genetically defined less active CX3CL1/CX3CR1 pathway is associated with a reduced risk of atherosclerotic disease in humans and the blockade of the CX3CL1/CX3CR1 pathway ameliorates the severity of atherosclerosis [124, 125]. Moreover, insulin resistance (IR) increases atherosclerotic lesion vulnerability, and this is related to the augment of CX3CL1/CX3CR1 axis [126]. All these indicate that any pharmacological agent that alters CX3CL1 signaling in AD should take into account any other potential effects.
